# Disease-Modifying Drug Uptake and Health Service Use in the Ageing MS Population

**DOI:** 10.3389/fimmu.2021.794075

**Published:** 2022-01-13

**Authors:** Huah Shin Ng, Jonas Graf, Feng Zhu, Elaine Kingwell, Orhan Aktas, Philipp Albrecht, Hans-Peter Hartung, Sven G. Meuth, Charity Evans, John D. Fisk, Ruth Ann Marrie, Yinshan Zhao, Helen Tremlett

**Affiliations:** ^1^ Department of Medicine, Division of Neurology and the Djavad Mowafaghian Centre for Brain Health, University of British Columbia, Vancouver, BC, Canada; ^2^ Department of Neurology, Medical Faculty, University Hospital, Heinrich-Heine-University, Düsseldorf, Germany; ^3^ Research Department of Primary Care & Population Health, University College London, London, United Kingdom; ^4^ Brain and Mind Centre, University of Sydney, Sydney, NSW, Australia; ^5^ Department of Neurology, Medical University of Vienna, Vienna, Austria; ^6^ Department of Neurology, Palacky University in Olomouc, Olomouc, Czechia; ^7^ College of Pharmacy and Nutrition, University of Saskatchewan, Saskatoon, SK, Canada; ^8^ Nova Scotia Health Authority and the Departments of Psychiatry, Psychology and Neuroscience, and Medicine, Dalhousie University, Halifax, NS, Canada; ^9^ Department of Internal Medicine, Max Rady College of Medicine, Rady Faculty of Health Sciences, University of Manitoba, Winnipeg, MB, Canada; ^10^ Department of Community Health Sciences, Max Rady College of Medicine, Rady Faculty of Health Sciences, University of Manitoba, Winnipeg, MB, Canada

**Keywords:** ageing, cohort studies, disease-modifying drugs, health services, hospitalization, multiple sclerosis, physician services

## Abstract

**Background:**

Evidence regarding the efficacy or effectiveness of the disease-modifying drugs (DMDs) in the older multiple sclerosis (MS) population is scarce. This has contributed to a lack of evidence-based treatment recommendations for the ageing MS population in practice guidelines. We examined the relationship between age (<55 and ≥55 years), DMD exposure and health service use in the MS population.

**Methods:**

We conducted a population-based observational study using linked administrative health data from British Columbia, Canada. We selected all persons with MS and followed from the most recent of their first MS or demyelinating event, 18^th^ birthday or 01-January-1996 (index date) until the earliest of emigration, death or 31-December-2017 (study end). We assessed DMD exposure status over time, initially as any versus no DMD, then by generation (first or second) and finally by each individual DMD. Age-specific analyses were conducted with all-cause hospitalizations and number of physician visits assessed using proportional means model and negative binomial regression with generalized estimating equations.

**Results:**

We included 19,360 persons with MS (72% were women); 10,741/19,360 (56%) had ever reached their 55th birthday. Person-years of follow-up whilst aged <55 was 132,283, and 93,594 whilst aged ≥55. Any DMD, versus no DMD in the <55-year-olds was associated with a 23% lower hazard of hospitalization (adjusted hazard ratio, aHR0.77; 95%CI 0.72-0.82), but not in the ≥55-year-olds (aHR0.95; 95%CI 0.87-1.04). Similar patterns were observed for the first and second generation DMDs. Exposure to any (versus no) DMD was not associated with rates of physician visits in either age group (<55 years: adjusted rate ratio, aRR1.02; 95%CI 1.00-1.04 and ≥55 years: aRR1.00; 95%CI 0.96-1.03), but variation in aRR was observed across the individual DMDs.

**Conclusion:**

Our study showed beneficial effects of the DMDs used to treat MS on hospitalizations for those aged <55 at the time of exposure. In contrast, for individuals ≥55 years of age exposed to a DMD, the hazard of hospitalization was not significantly lowered. Our study contributes to the broader understanding of the potential benefits and risks of DMD use in the ageing MS population.

## Introduction

Multiple sclerosis (MS) is a chronic, immune-mediated disease characterized by demyelination and neurodegeneration affecting both brain and spinal cord. While most people will be diagnosed with MS between the ages of 20-50 years, the average age range of people living with MS in North America is between 55 and 65 years (1, 2). Despite this, people with MS aged 55 years or older have often been excluded from clinical trials testing the efficacy of disease-modifying drugs (DMDs) (1–4). While short-term MS clinical trials showed limited benefits of taking DMD after age 53 years (5), and the potential for harm (e.g., higher neoplasm risk, especially after age 45 years) (6), all based on meta-analyses, the evidence regarding the long-term efficacy or effectiveness and safety profile of DMDs in the older MS population is scarce (7, 8). This has contributed to a lack of treatment recommendations for the ageing MS population in practice guidelines (3, 4).

Health administrative data which captures health care use information offers opportunity to assess the real-world effectiveness of the DMDs used to treat MS. This approach has been successfully applied to examine the safety and effectiveness of the DMDs in the general, or healthcare insured, MS population (9–13).

In this study, we accessed population-based health administrative data captured over a 22-year period in the province of British Columbia, Canada, to examine the relationship between age (<55 and ≥55 years), DMD exposure and health service use in the MS population. We examined age as a dynamic process for each person, by dividing the individual’s time spent before and after reaching age 55 years.

## Materials and Methods

### Data Sources

We conducted a population-based observational study using linked administrative health data. These prospectively collected data covered the population of British Columbia, comprising 4.64 million residents, and representing around 13% of the Canadian population (14). The linked data comprised five datasets: (i) the provincial health insurance registry (15) providing demographic information for each individual, including sex, date of birth, residency status and location (three digit postal codes); (ii) vital statistics data (16) providing the date of death; (iii and iv) physician billing (17) and discharge abstract databases (18) capturing all physician visits and hospitalizations, with reasons for the visit or admission coded using the International Classification of Diseases (ICD)-9/10 system; and (v) the provincial prescription database (PharmaNet) (19) capturing all prescription drugs dispensed at outpatient and community pharmacies.

### Study Population

We selected all persons with MS by using a validated algorithm with the cases defined as having at least 3 MS diagnostic codes (ICD-9/10 340/G35) in the hospital and/or physician data or an MS DMD record in the prescription data, as outlined previously (20–22). We assigned an index date to each person based on the most recent date of: the first MS or demyelinating event recorded in the hospital, physician or prescription data (**Supplementary Tables 1**, **2**), or 01-January-1996 (the date when the provincial prescription data first became available), or the person’s 18^th^ birthday. All persons required at least one year of residency in British Columbia before the index date, and were followed from the index date until the study end date defined as the earliest of emigration from the province, death or 31-December-2017.

We determined the cohort characteristics at the index date, including age, calendar year, sex, and socioeconomic status (reported as neighborhood-level income quintiles according to a person’s three-digit postal codes by linkage to census data) (23). The burden of comorbidity was measured using a modified Charlson Comorbidity Index based on the diagnoses captured in the hospital and physician data during the one-year before the index date, with hemiplegia and paraplegia excluded to avoid misclassifying symptoms related to MS as comorbidity (24, 25).

### DMD Exposure

The DMDs available during our study period (**Supplementary Table 2**) included the first generation DMDs – beta-interferon and glatiramer acetate, and the second generation DMDs – natalizumab, fingolimod, dimethyl fumarate, teriflunomide, alemtuzumab, daclizumab, and ocrelizumab. We grouped all beta-interferon products together as one class. We assessed DMD exposure status as a time-varying variable, initially as any versus no DMD, then by generation (first or second), and finally by individual DMDs. Neither daclizumab nor ocrelizumab were included in the assessment of individual DMDs due to the small number of individuals (<6) exposed over the study period, preventing the derivation of reliable estimates.

We determined the DMD exposure periods according to the number of days supplied for each individual DMD. A DMD was considered as being discontinued when there were no further dispensations for the DMD for greater than 90 days. The discontinuation date was defined as the last DMD prescription fill date plus the number of days supplied, with a 30-day grace period applied (26). The exception was for alemtuzumab and ocrelizumab, whereby periods of exposure were defined as one year (for alemtuzumab) and six months (for ocrelizumab), from the date of first supply, plus a 30-day grace period if no further DMD fills occurred. Finally, as persons could not be on more than one DMD at the same time, once a person filled a new DMD prescription, then the previous DMD was considered discontinued. DMD exposure was assessed as a time-varying variable (i.e. a person’s DMD exposure status was allowed to change over time).

### Age Grouping

We assessed each person’s current age as a time-varying variable, grouped as <55 or ≥55 years old. This age grouping was selected partly because older individuals (≥55 years) have often been excluded from enrolling in MS clinical trials (1–4), and partly because the prevalence of persons with MS aged 55 and above has risen in recent years (20, 27), such that these individuals represent a growing yet understudied group.

### Outcomes

The outcome measures were all-cause hospitalizations and number of physician visits.

Hospitalization data included day surgery/minor procedures (but not drug infusions e.g., for natalizumab or alemtuzumab) (28). For the hospitalizations, any overlapping admissions or any new admission that occurred within one day of the previous hospitalization were counted as a single event (10, 29). For the physician visits, multiple claims with the same primary ICD code captured on the same day were counted as a single visit (10, 29). Neurologist visits were also excluded from the count as the number of these visits was anticipated to be higher in persons exposed to a DMD (versus no DMD) as part of routine care (4) (other physician specialties cannot prescribe an MS DMD in British Columbia). In addition, any pregnancy-related encounters (hospitalizations or physician visits based on the primary ICD code) were not included as an outcome as DMD cessation was expected to be common during pregnancy (4).

### Statistical Analyses

We described the cohort characteristics at the index date by age group (<55 versus ≥55 years old at the index date) and DMD exposure (at any time during follow-up), using counts and percentages for the categorical variables, and means and standard deviations for the continuous variables. Person-years of follow-up by current age group at the time of exposure for each individual DMD was also reported.

We included an interaction term between DMD exposure and current age group (<55 or ≥55 years) at the time of follow-up to estimate the age-specific associations between DMD exposure and outcomes (all-cause hospitalizations and the number of physician visits). For each analysis, the period of ‘no DMD exposure’ was used as the reference category.

All-cause hospitalizations were assessed using proportional means models with robust sandwich variance estimates (30). Models were adjusted for sex, socioeconomic status (quintiles), age (continuous) and calendar year (continuous) at the index date, and for the Charlson comorbidity score, categorized as: 0, 1, 2, ≥3, and updated annually over time. The period of hospitalization was discounted from the follow-up time as a person could not be at risk of another hospitalization during the existing hospital stay. Findings were expressed as adjusted hazard ratios (aHRs) with the corresponding 95% confidence intervals (CIs).

The number of physician visits were examined using negative binomial regression models fitted by generalized estimating equations with an exchangeable working correlation matrix (31). The number of physician visits were calculated annually, or by DMD exposure periods when there were changes in DMD status within a year. An offset was included in the model to account for the variable length of time periods (log of person-time). Models were adjusted for sex and socioeconomic status (quintiles) at the index date, and the following covariates over time (updated on an annual basis) including age (continuous), Charlson comorbidity score (categorized as: 0, 1, 2, ≥3) and calendar year (continuous; to account for any secular changes in healthcare use). Findings were expressed as adjusted rate ratios (aRRs) with the corresponding 95% CIs.

A complementary analysis was also performed to describe the DMD exposure status for any person who had reached age 55 years at any time before the study end date.

We conducted the statistical analyses using SAS software version 9.4 (SAS Institute, Cary, NC, USA).

### Study Registration and Ethical Approval

This study was registered with ClinicalTrials.gov (NCT04472975). We obtained ethics approval from the University of British Columbia’s Clinical Research Ethics Board (H18-00407).

## Results

### Cohort Characteristics

We identified a total of 19,360 persons with MS (72.0% were women). Almost half (44.1%, 8,533/19,360) of the cohort entered the study between 1996-1999, with the remainder entering between 2000-2017. The mean follow-up time was 11.7 years (SD 7.3). At the index date, 78.7% (15,235/19,360) were aged <55 years and 21.3% (4,125/19,360) were ≥55 years (**Table 1**). Of those aged <55 years at the index date, 29.7% (4,526/15,235) filled a DMD prescription during follow-up, whereas 5.0% (206/4,125) of persons aged ≥55 years at the index date did so. Within both age groups, those treated with an MS DMD were approximately 4-5 years younger relative to those who were untreated. For example, for those under age 55 years at the index date, the mean age was 36.1 years [SD 8.7] for those ever DMD exposed during follow-up, versus 40.5 years [SD 8.9] for those unexposed.

**Table 1:** Characteristics of the multiple sclerosis study population by age group at the index date (<55 versus ≥55 years old) and by exposure to a disease-modifying drug at any time during follow-up, n=19,360.

Characteristics	Age at Index Date <55 Years, n=15,235		Age at Index Date ≥ 55 Years, n=4,125	
	DMD-Treated^a^ n=4,526	Not Treated^a^ n=10,709	DMD-Treated^a^ n=206	Not Treated^a^ n=3,919
**Sex, n (%)**				
Women	3,324 (73.4)	7,868 (73.5)	145 (70.4)	2,603 (66.4)
Men	1,202 (26.6)	2,841 (26.5)	61 (29.6)	1,316 (33.6)
**Age at index date in years,** mean (SD)	36.1 (8.7)	40.5 (8.9)	58.9 (4.6)	64.5 (8.0)
**Socioeconomic status** **^b^** **, n (%)**				
1 (lowest income quintile)	876 (19.4)	2,037 (19.0)	38 (18.4)	812 (20.7)
2	839 (18.5)	2,075 (19.4)	31 (15.0)	750 (19.1)
3	953 (21.1)	2,149 (20.1)	39 (18.9)	790 (20.2)
4	958 (21.2)	2,311 (21.6)	48 (23.3)	777 (19.8)
5 (highest income quintile)	888 (19.6)	2,083 (19.5)	50 (24.3)	758 (19.3)
Unavailable	12 (0.3)	54 (0.5)	<6	32 (0.8)
**Comorbidity score** **^c^** **, n (%)**				
0	3,820 (84.4)	8,552 (79.9)	154 (74.8)	2,525 (64.4)
1	553 (12.2)	1,585 (14.8)	35 (17.0)	806 (20.6)
2	121 (2.7)	388 (3.6)	11 (5.3)	335 (8.5)
≥ 3	32 (0.7)	184 (1.7)	6 (2.9)	253 (6.5)
**Calendar year at index date, n (%)**				
1996-1999	1,479 (32.7)	5,132 (47.9)	50 (24.3)	1,872 (47.8)
2000-2009	1,818 (40.2)	3,374 (31.5)	73 (35.4)	1,152 (29.4)
2010-2017	1,229 (27.2)	2,203 (20.6)	83 (40.3)	895 (22.8)
**Follow-up** **^a^** ** time in years,**				
median (Q1, Q3)	12.2 (5.9, 18.6)	12.0 (5.4, 20.0)	8.2 (3.9, 13.3)	8.6 (4.0, 14.7)
mean (SD)	12.2 (7.0)	12.2 (7.5)	9.3 (6.5)	9.7 (6.6)
**Number of different DMD prescriptions filled during the follow-up** ^a^				
1	2,865 (63.3)	N/A	171 (83.0)	N/A
2	1,194 (26.4)		30 (14.6)	
≥ 3	467 (10.3)		<6	
**First DMD prescription, n (%)**				
Beta-interferon^d^	2,833 (62.6)	N/A	122 (59.2)	N/A
Glatiramer acetate	1,080 (23.9)		48 (23.3)	
Natalizumab	63 (1.4)		<6	
Fingolimod	31 (0.7)		<6	
Dimethyl fumarate	300 (6.6)		13 (6.3)	
Teriflunomide	181 (4.0)		15 (7.3)	
Alemtuzumab	36 (0.8)		<6	
Daclizumab	<6		<6	
Ocrelizumab	<6		<6	
**Number of individuals ever exposed, by type of DMD, during follow-up** **^a^** **, n (%)**				
**First generation DMDs –any** **^e^**	3,953 (87.3)	N/A	171 (83.0)	N/A
Beta-interferon^d^	3,016 (66.6)		124 (60.2)	
Glatiramer acetate	1,655 (36.6)		64 (31.1)	
**Second generation DMDs – any** **^e^**	1,703 (37.6)		53 (25.7)	
Natalizumab	277 (6.1)		9 (4.4)	
Fingolimod	416 (9.2)		<6	
Dimethyl fumarate	736 (16.3)		22 (10.7)	
Teriflunomide	497 (11.0)		23 (11.2)	
Alemtuzumab	178 (3.9)		<6	
Daclizumab	6 (0.1)		<6	
Ocrelizumab	<6		<6	

Key: SD, standard deviation; DMD, disease-modifying drug, N/A, not applicable.

As per data privacy and access agreements, small cell size (<6 individuals within any group) are suppressed.

a Follow-up was from index date until the study end date (up to December 31st 2017).

b Socioeconomic status is reported by neighborhood income quintiles according to a person’s three-digit postal codes (closest available to the index date).

c Comorbidity was measured using the modified Charlson Comorbidity Index (exclude hemiplegia/paraplegia to avoid misclassifying MS complications as comorbidity) based on the diagnoses captured in the hospital and physician data during the one-year before the index date.

d All beta-interferon products were grouped together as one class.

e Some people were exposed to >1 DMD; hence the sum of the individual first or second generation DMDs exceeds the sum of any first or second generation DMD.

Irrespective of DMD exposure status during follow-up, the comorbidity burden (measured using the modified Charlson Comorbidity Index) was lower for persons <55 years at the index date, relative to those ≥55 years old. With respect to DMD exposure status, the comorbidity burden at the index date was lower in the DMD-treated group compared to the non-treated group. For example, for those age ≥55 years at the index date, 52/206 (25.2%) had a comorbidity in the DMD-treated group, versus 1,394/3,919 (35.6%) in the non-treated group. The socio-economic quintiles were generally evenly distributed across all four groups (**Table 1**).

Of those ever filling a DMD prescription during follow-up, the proportion exposed to a first generation DMD was similar regardless of age at the index date. Whereas, a higher proportion of the younger (<55 years) MS cases were exposed to a second generation DMD (37.6%, 1,703/4,526), compared to older individuals (≥55 years; 25.7%, 53/206). A higher proportion of younger persons with MS (<55 years at the index date) also switched between DMDs at least once during follow-up (36.7%; 1,661/4,526), compared to older individuals (≥55 years; 17.0%, 35/206).

As younger persons (<55 years) could transition to the older age group (≥55 years) throughout our >20-year study period, an overview of the person-years of follow-up by each person’s current age group and DMD exposure status is shown in **Table 2**. Not unexpectedly, the number of person-years of follow-up was higher in persons with a current age of <55 (versus ≥55 years), irrespective of DMD exposure status. Of note the person-years of follow-up for certain DMDs were modest particularly in the older (≥55 years) age group.

**Table 2:** Person-years of follow-up in the multiple sclerosis cohort by each person’s current age, grouped as <55 or ≥55 years old, and by disease-modifying drug exposure status.

Person-Years of Follow-Up	Person’s Current Age	
	<55 Years [1]	≥55 Years [2]
**During periods of exposure to:**		
**Any DMDs**	20,555.5	4,414.8
***Any first generation DMDs***	17,180.4	3,842.8
Beta-interferon^a^	12,413.7	2,911.9
Glatiramer acetate	4,766.6	930.8
***Any second generation DMDs***	3,375.2	572.0
Natalizumab	745.6	85.5
Fingolimod	871.4	115.6
Dimethyl fumarate	1,051.0	195.4
Teriflunomide	489.0	168.5
Alemtuzumab	216.2	6.4
Daclizumab	<6	<6
Ocrelizumab	<6	<6
**No DMD**	111,727.6	89,179.3
**Total person-years of follow-up**	132,283.1	93,594.1

Key: DMD, disease-modifying drug.

a All beta-interferon products were grouped together as one class.

Total cohort size=19,360. Of these, by the study end n=10,741/19,360 (55.5%) had ever reached their 55^th^ birthday, with n=4,125/10,741 (38.4%) doing so by the index date and n=6,616/10,741 (61.6%) during follow-up. The remainder, n=8,619/19,360 (44.5%) never reached their 55th birthday by the study end. Thus, n=6,616 individuals contributed follow-up time to both columns [1] and [2], n=8,619 only to column [1] and n=4,125 only to column [2].

### Hospitalizations

**Figure 1:**
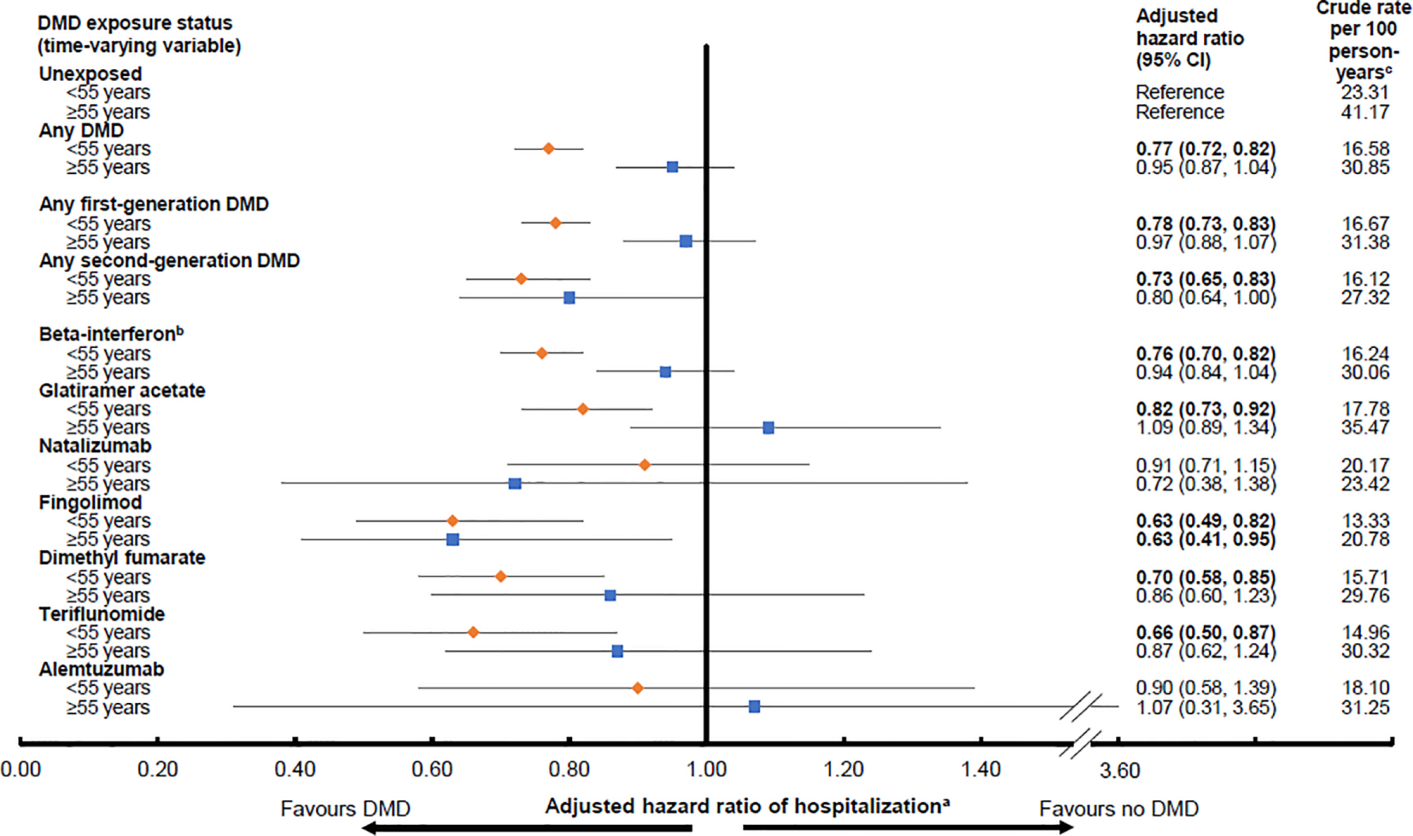
Exposure to a disease-modifying drug for multiple sclerosis and hazard of hospitalization by age group (<55 or ≥55 years old). Key: CI, confidence interval; DMD, disease-modifying drug. Bold indicates p<0.05. ^a^Results were adjusted for sex, socioeconomic status (quintiles), age (continuous) and calendar year (continuous) at the index date, and for Charlson comorbidity score (categorical: 0, 1, 2, ≥3) over time (updated on annual basis). Hazard ratios were estimated by introducing interaction terms between current age group (<55 versus ≥55 years at the time of exposure) and the DMD exposure variables shown in the Figure. ^b^All beta-interferon products were grouped together as one class. ^c^Person-years of follow-up for the calculation of crude rate were as per except that the duration of a hospitalization was discounted from the follow-up time.

Any DMD, relative to no DMD, was associated with a 23% lower hazard of hospitalization for those aged <55 years at the time of DMD exposure (aHR 0.77; 95%CI 0.72-0.82), but was not for those aged ≥55 years (aHR 0.95; 95%CI 0.87-1.04), **Figure 1**. Similar trends were observed for the first generation DMDs, where a 22% significantly lower hazard was observed for those aged <55 years, and, for the second generation DMDs, a 27% significantly lower hazard. Neither of these findings reached significance for those aged ≥55 years at the time of DMD exposure, where the lower hazard ranged from 3% to 20%. When the DMDs were assessed individually, the hazard of hospitalization for those aged <55 years at the time of exposure ranged from a 9% lower hazard for natalizumab, to 10% for alemtuzumab, 18% for glatiramer acetate, 24% for beta-interferon, 30% for dimethyl fumarate, 34% for teriflunomide, and 37% for fingolimod. All reached statistical significance, except for natalizumab and alemtuzumab, although the 95% confidence intervals were also very wide for these two DMDs. For person’s aged ≥55 years at the time of DMD exposure, the corresponding HRs did not reach significance, except for fingolimod, which was associated with a 37% lower hazard of hospitalization. All results for the DMD exposure and hospitalizations analyses by age group are shown in **Figure 1**.

### Physician Visits

**Figure 2:**
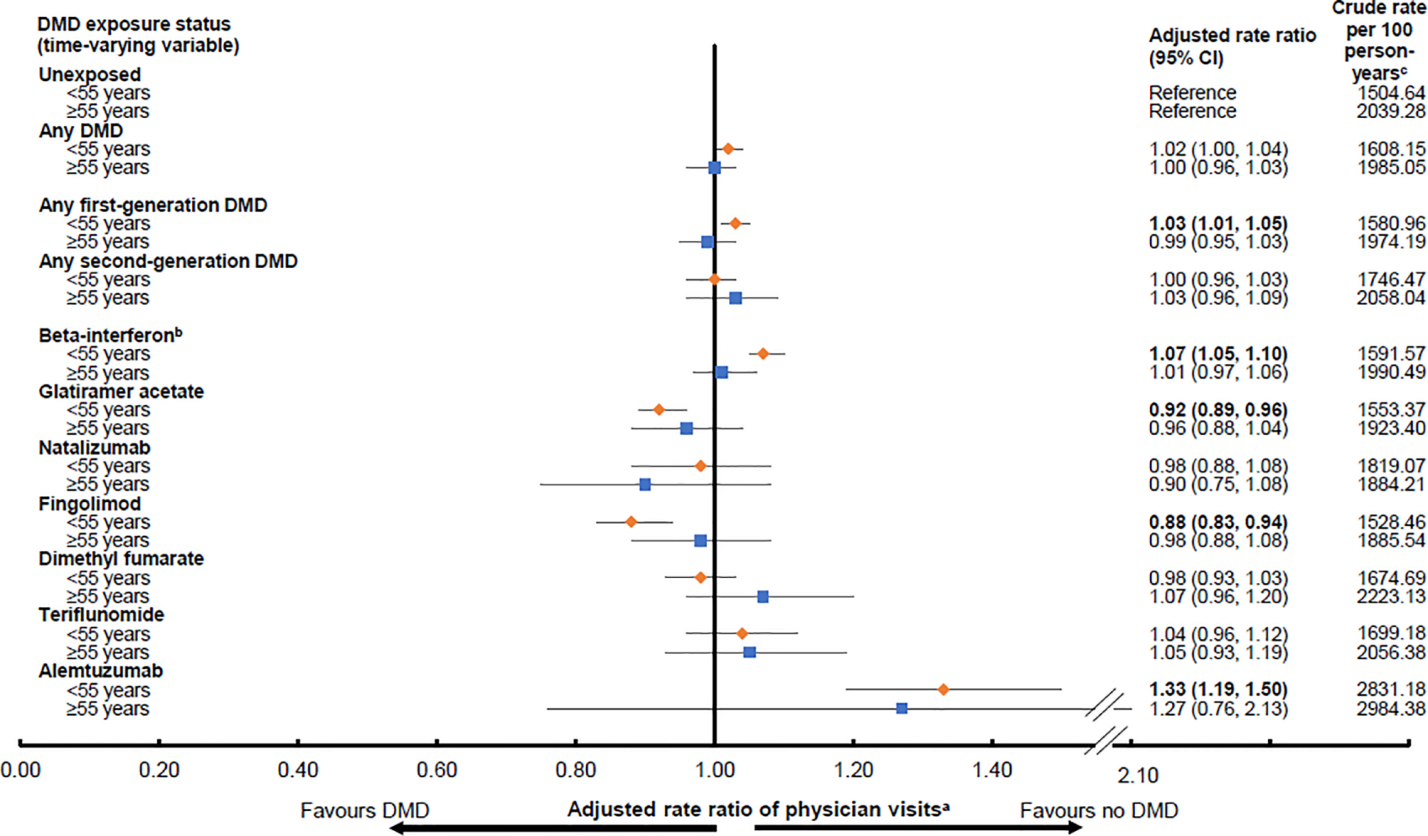
Exposure to a disease-modifying drug for multiple sclerosis and rates of physician visits^d^ by age group (<55 or ≥55 years old). Key: CI, confidence interval; DMD, disease-modifying drug. Bold indicates p<0.05. ^a^Results were adjusted for sex and socioeconomic status (quintiles) at the index date, and the following characteristics over time on a yearly basis: age (continuous), calendar year (continuous), and Charlson comorbidity score (categorical: 0, 1, 2, ≥3). Rate ratios were estimated by introducing interaction terms between current age group (<55 versus ≥55 years at the time of exposure) and the DMD exposure variables shown in the Figure. ^b^All beta-interferon products were grouped together as one class. ^c^Person-years of follow-up are shown in **Table 2** and were used to calculate the crude rates. ^d^As outlined in the study methods, neurologist visits were excluded, as were pregnancy-related visits.

While exposure to any DMD (versus no DMD) was not associated with altered rates of physician visits in either age group (<55 years: aRR 1.02; 95%CI 1.00-1.04 and ≥55 years: aRR 1.00; 95%CI 0.96-1.03), variation was observed across the individual DMDs (**Figure 2**). A 27-33% higher rate of physician visits was observed during exposure to a second generation DMD, alemtuzumab, reaching significance in the younger, but not older population. In contrast, exposure to another second generation DMD, fingolimod was associated with a significantly lower rate of physician visits, by 12%, for those <55 years. More modest differences were seen for the first generation DMDs, but for those aged <55 years, with beta-interferon associated with a 7% higher rate of physician visits and glatiramer acetate with an 8% lower rate. All results for the DMD exposure and physician visits analyses by age group are shown in **Figure 2**.

### Complementary Analysis

By the study end 10,741 persons had ever reached their 55^th^ birthday, with 38.4% (n=4,125/10,741) doing so by the index date and 61.6% (n=6,616/10,741) during follow-up. Approximately 15% (n=1,657/10,741) were exposed to a DMD at any time point during follow-up (**Supplementary Table 3**). While 12% (n=1,302/10,741) had their first DMD before age 55 years, nearly half of these (n=596/1,302) were no longer taking DMD once aged ≥55 years. In total, over 3% (n=355/10,741) of persons initiated their first DMD at the age of 55 years or older.

## Discussion

We assessed the effect of current age on the association between DMD exposure and health service utilization in a population-based MS cohort with over 200,000 person-years of follow-up, and all within a universal healthcare setting. Exposure to any DMD or to any first generation DMD (versus no DMD) was associated with a 22-23% lower hazard of hospitalization for those aged <55 years at the time of exposure, while exposure to any second generation DMD was associated with a 27% lower hazard of hospitalization. In contrast, in older adults (≥55 years), DMD exposure, whether assessed as any DMD or any first or second generation or even by individual DMDs (versus no DMD), was generally not associated with a lower risk of hospitalization. Finally, while regardless of age (<55 or ≥55 years), exposure to any DMD, relative to no DMD, was not associated with an altered rate of physician visits, considerable variation was observed across the individual DMDs. Our findings offer insights into the effects of ageing on the relationship between the DMDs used to treat MS and health services in the real-world setting.

While several studies have assessed the relationship between the MS DMDs and healthcare utilization (9–12), we were unable to find another study to compare our age-specific findings. A study from the United States co-authored by a pharmaceutical manufacturer of an MS DMD examined patterns of healthcare utilization in 28,427 persons with MS by insurance type (commercial versus the federal insurance plan, Medicare Advantage) within different age groups (32). However, the study was cross-sectional in design, spanning just one-year and was restricted to persons under 65 years of age. The authors found that MS persons with a federal insurance plan had a significantly higher mean count of all-cause inpatient and ambulatory visits compared to those with a commercial insurance plan. While findings were consistent across age groups, at least once age 30 years was reached, the authors did not report which individual DMDs contributed to these findings (32). Efforts have also been made to examine the effects of ageing on the efficacy of DMDs by using data from clinical trials. However, there are some limitations and challenges with these studies. One example is a meta-analysis which included 26 clinical trials of 14 different DMDs published between 1995 and 2019, and comprised 28,082 relapsing-remitting MS persons. Authors found no statistically significant associations between age and reduction(s) in disease activity (measured as annualized relapse rates and magnetic resonance imaging metrics, such as gadolinium-enhancing lesions and T2 lesions) when the DMD- and placebo-treated groups were compared (33). However, as individual-level data were inaccessible, this meta-analysis had to rely on group-level average ages as reported within each clinical trial to examine potential age-related differences in DMD efficacy (33). Moreover, the original clinical trials were not designed to examine DMD efficacy in the ageing population and individuals older than 55 years were excluded from enrollment, such that the average ages of participants ranged from 33 to 40 years (33).

The differences we found in the association between DMD exposure and the hazard of hospitalizations by age group (<55 versus ≥55 years old) does concur with broader observations from both natural history studies of MS and the MS clinical trials. For example, clinical trials have demonstrated beneficial effects of DMDs on reducing or preventing MS relapses in the short-term (34), which may in turn lower the risk of hospitalization (9, 35–38). However, the frequency of MS relapses naturally decreases over time and with age (39, 40), being less common in older individuals, particularly after 60 years of age (2). Furthermore, the DMDs appear less efficacious and less effective in progressive MS (primary or secondary) (41–43) and a higher proportion of the older MS population will have progressive MS (1, 2, 39). The longer-term effects of the DMDs in preventing disability associated with disease progression and ageing is uncertain (2). For example, a study conducted in British Columbia, Canada, showed that exposure to beta-interferon (versus no exposure) was not associated with a lower hazard of reaching an Expanded Disability Status Scale [EDSS] score of 6 in older MS adults, aged≥50 years (44). Further, a meta-analysis, which included 38 clinical trials of 13 different DMDs with over 28,000 MS persons, showed that the effects of DMDs on MS disability progression was strongly dependent on age, with limited benefits of receiving DMDs after age 53 years (5).

In our study, we found that exposure to any DMD was not associated with differences in the rate of physician visits by age group (<55 versus ≥55 years old), although variation across individual DMDs was observed, especially in the younger age group (<55 years). Use of the MS DMDs typically requires regular laboratory testing and safety-related monitoring. Therefore, it is possible that any potential benefits on, or decreases in, physician visits or hospitalizations related to an anticipated benefit of the DMDs on disease activity may be offset by the increase in safety-related monitoring (10). Higher rates of physician visits, ranging from 27-33%, were observed in both age groups (<55 and ≥55 years old at the time of exposure) while exposed to alemtuzumab, although this failed to reach significance in the older age group. Alemtuzumab requires regular monthly monitoring due to the risk of adverse events, such as autoimmune disorders, which may contribute to these higher rates (45). Similarly, exposure to beta-interferon, which requires regular monitoring for liver and thyroid function (46), was associated with a higher rate of physician visits. However, this was a much more modest 7% and only demonstrated in the younger age group (<55 years old at the time of exposure). In contrast, exposure to glatiramer acetate, which requires no formal laboratory testing (47), was associated with an 8% lower rate of physician visits, and exposure to fingolimod was associated with a 12% lower rate (again in the <55 year old age group only), despite the necessity of regular biochemical liver testing (48). Fingolimod is generally reserved as a second-line therapy in Canada, and a lower rate of physician visits may be due to decreases in disease activity.

### Complementary Descriptive Analysis - DMD Use in the Older Age Group

Currently, it remains unclear if DMD treatment should be continued (or even started) in the MS population aged 55 years or older. To address this, a large phase 4 randomized controlled DMD treatment discontinuation trial is currently underway in the Unites States which includes persons ≥55 years old, and its findings may add crucial knowledge to this emerging aspect of MS care (49). While, as expected, a smaller proportion of our older (versus younger) persons with MS were DMD exposed (22, 32, 50), we also observed that the proportions of persons continuing or discontinuing DMD were similar. Specifically, the proportions of persons who initiated their first DMD before age 55 years, and who continued or discontinued once aged ≥55 years were rather similar (n=706/1,302 versus n=596/1,302, respectively) (**Supplementary Table 3**). These findings may reflect individual differences in diseases severity and/or the lack of clear treatment guidelines for the older patient population in clinical practice.

### Strengths and Limitations

Our study has both strengths and limitations. Given that some of the newer second-generation DMDs only became widely available towards the end of our study, the total person-years exposed was modest for certain DMDs, particularly in the older (≥55 years) age group. Therefore, we could not examine the specific causes of hospitalizations due to modest event rates by age group which would have hindered derivation of reliable estimates. It is plausible that older individuals are at higher risk of being hospitalized for non-MS than MS-related causes (51). We cannot exclude that clinical or other characteristics that we did not have access to in our administrative data, such as MS disease course, disability level, or cognitive status, or lifestyle factors, such as smoking and alcohol consumption and/or ancestry/ethnicity may have influenced our findings. It remains possible that other confounders not available to us could also be of relevance. However, we were able to adjust for sex, socioeconomic status, and comorbidity burden over time. In addition, we were able to account for the changing treatment status of persons over time by using a longitudinal approach when examining DMD exposure. We consider the potential for selection bias in our study to be minimal given the universal healthcare setting, and our access to comprehensive health care data for all residents of the province, irrespective of ability to pay. Furthermore, we used a validated case definition in our study to select persons with MS. Other strengths of our study are the use of objectively collected population-based data, including linked health administrative information, and the long duration of follow-up (mean 11.7 years).

## Conclusions

Our findings suggest that use of the DMDs to treat MS may be more effective in preventing hospitalizations in younger persons (aged <55 years), compared to older individuals (aged ≥55 years). For those aged <55 years, similar trends were observed for both the first and second generation DMDs. In contrast, a more varied and complex picture evolved when the relationship between DMD exposure and physician visits was examined in the older and younger age groups. This might, in part, reflect the different safety-related monitoring strategies required for the different DMDs. Further studies are warranted in order to expand treatment guidelines for an ageing MS population.

## Data Availability Statement

The data analyzed in this study is subject to the following licenses/restrictions: As we are not the data custodians, we are not authorized to make the data available. With the appropriate approvals, the data may be accessed through the Population Data British Columbia. Requests to access these datasets should be directed to Population Data British Columbia.

## Ethics Statement

The studies involving human participants were reviewed and approved by the University of British Columbia’s Clinical Research Ethics Board (H18-00407). Written informed consent for participation was not required for this study in accordance with the national legislation and the institutional requirements.

## Author Contributions

HN, JG, and HT interpreted the results and drafted the manuscript. HN, JG, FZ, EK, CE, JF, RM, YZ, and HT conceptualized and designed the study. FZ, EK, CE, JF, RM, YZ, and HT facilitated obtaining funding (PI: Tremlett, CIHR Project and Foundation award). HN, JG, and FZ performed data analysis. All authors revised the manuscript critically for intellectual content, approved the final version to be published, and agreed to be accountable for all aspects of the work.

## Funding

This study was supported by the Canadian Institutes of Health Research (CIHR) Project and Foundation grant (PJT-156363 and FDN-159934, PI: Tremlett).

## Author Disclaimer

All inferences, opinions, and conclusions drawn in this manuscript are those of the authors, and do not reflect the opinions or policies of the British Columbia Data Steward(s).

## Conflict of Interest

HN receives funding from the Multiple Sclerosis Society of Canada’s endMS Postdoctoral Fellowship, and the Michael Smith Foundation for Health Research Trainee Award. During the past year, HSN has received funding from the Canadian Institutes of Health Research (CIHR) Drug Safety and Effectiveness Cross-Disciplinary Training Program. JG has received in the last 3 years travel/meeting/accommodation reimbursements from Merck Serono, Sanofi-Genzyme, Grifols, and receives a Research Fellowship from the Deutsche Forschungsgemeinschaft (project number 438899010, GZ: GR 5665/1-1). OA reports research funding from: German Research Foundation (DFG), German Ministry of Science (BMBF), German National MS Society (DMSG-LV), Biogen and Novartis; personal fees from Alexion, Biogen, EMD, Novartis, Roche, and Teva. PA reports research funding and personal fees from: European Fund for Regional Development (EFRE), Allergan, Celgene, Biogen, Ipsen, Merck, Merz, Roche; personal fees from Janssen Cilag, Lilly, and Teva. H-PH received outside this work, with approval of the Rector of Heinrich-Heine University and the CEO of University of Düsseldorf Hospital honoraria for consulting, serving on steering committees and speaking from Alexion, Bayer Healthcare, Biogen, Celgene BMS, Geneuro, LFB, Medimmune, Merck Serono, Novartis, Octapharma, Roche, Sanofi Genzyme, TG Therapeutics and VielaBio. SM received honoraria for lecturing and travel expenses for attending meetings from Almirall, Amicus Therapeutics Germany, Bayer Health Care, Biogen, Celgene, Diamed, Genzyme, MedDay Pharmaceuticals, Merck Serono, Novartis, Novo Nordisk, ONO Pharma, Roche, Sanofi-Aventis, Chugai Pharma, QuintilesIMS, and Teva and his research is funded by the German Ministry for Education and Research (BMBF), Deutsche Forschungsgemeinschaft (DFG), Else Kröner Fresenius Foundation, German Academic Exchange Service, Hertie Foundation, Interdisciplinary Center for Clinical Studies (IZKF) Muenster, German Foundation Neurology, and by Almirall, Amicus Therapeutics Germany, Biogen, Diamed, Fresenius Medical Care, Genzyme, Merck Serono, Novartis, ONO Pharma, Roche, and Teva. CE receives funding from CIHR. JF receives research funding from: CIHR, Multiple Sclerosis Society of Canada, Crohn’s and Colitis Canada, Research Nova Scotia; consultation and distribution royalties from MAPI Research Trust. RM receives research funding from: CIHR, Research Manitoba, Multiple Sclerosis Society of Canada, Multiple Sclerosis Scientific Foundation, Crohn’s and Colitis Canada, National Multiple Sclerosis Society, CMSC and the US Department of Defense, and is a co-investigator on studies receiving funding from Biogen Idec and Roche Canada. HT is the Canada Research Chair for Neuroepidemiology and Multiple Sclerosis. Current research support received from the National Multiple Sclerosis Society, the Canadian Institutes of Health Research, the Multiple Sclerosis Society of Canada and the Multiple Sclerosis Scientific Research Foundation. In addition, in the last five years, has received research support from the UK MS Trust; travel expenses to present at CME conferences from the Consortium of MS Centres (2018), the National MS Society (2016, 2018), ECTRIMS/ACTRIMS (2015, 2016, 2017, 2018, 2019, 2020), American Academy of Neurology (2015, 2016, 2019). Speaker honoraria are either declined or donated to an MS charity or to an unrestricted grant for use by HT’s research group.

The remaining authors declare that the research was conducted in the absence of any commercial or financial relationships that could be construed as a potential conflict of interest.

## Publisher’s Note

All claims expressed in this article are solely those of the authors and do not necessarily represent those of their affiliated organizations, or those of the publisher, the editors and the reviewers. Any product that may be evaluated in this article, or claim that may be made by its manufacturer, is not guaranteed or endorsed by the publisher.

## References

[1] Awad A Stüve O. Multiple Sclerosis in the Elderly Patient. Drugs Aging (2010) 27:283–94. doi: 10.2165/11532120-000000000-00000 20359260

[2] Vaughn CB Jakimovski D Kavak KS Ramanathan M Benedict RHB Zivadinov R. Epidemiology and Treatment of Multiple Sclerosis in Elderly Populations. Nat Rev Neurol (2019) 15:329–42. doi: 10.1038/s41582-019-0183-3 31000816

[3] Rae-Grant A Day GS Marrie RA Rabinstein A Cree BAC Gronseth GS. Comprehensive Systematic Review Summary: Disease-Modifying Therapies for Adults With Multiple Sclerosis. Neurology (2018) 90:789–800. doi: 10.1212/WNL.0000000000005345 29686117

[4] Rae-Grant A Day GS Marrie RA Rabinstein A Cree BAC Gronseth GS. Practice Guideline Recommendations Summary: Disease-Modifying Therapies for Adults With Multiple Sclerosis. Report of the Guideline Development, Dissemination, and Implementation Subcommittee of the American Academy of Neurology. Neurology (2018) 90:777–88. doi: 10.1212/WNL.0000000000005347 29686116

[5] Weideman AM Tapia-Maltos MA Johnson K Greenwood M Bielekova B. Meta-Analysis of the Age-Dependent Efficacy of Multiple Sclerosis Treatments. Front Neurol (2017) 8:577. doi: 10.3389/fneur.2017.00577 29176956 PMC5686062

[6] Prosperini L Haggiag S Tortorella C Galgani S Gasperini C. Age-Related Adverse Events of Disease-Modifying Treatments for Multiple Sclerosis: A Meta-Regression. Mult Scler (2021) 27:1391–402. doi: 10.1177/1352458520964778 33104449

[7] Zanghì A Avolio C Amato MP Filippi M Trojano M Patti F. First-Line Therapies in Late-Onset Multiple Sclerosis: An Italian Registry Study. Eur J Neurol (2021) 28:4117–23. doi: 10.1111/ene.15006 PMC9291454 34216532

[8] D’Amico E Patti F Zanghì A Chisari CG Lo Fermo S Zappia M. Late-Onset and Young-Onset Relapsing-Remitting Multiple Sclerosis: Evidence From a Retrospective Long-Term Follow-Up Study. Eur J Neurol (2018) 25:1425–31. doi: 10.1111/ene.13745 29956427

[9] Nicholas J Boster A Wu N Yeh W-S Fay M Kendter J. Comparison of Disease-Modifying Therapies for the Management of Multiple Sclerosis: Analysis of Healthcare Resource Utilization and Relapse Rates From US Insurance Claims Data. Pharmacoecon Open (2018) 2:31–41. doi: 10.1007/s41669-017-0035-2 29464673 PMC5820236

[10] Al-Sakran L Marrie RA Blackburn D Knox K Evans C. Association Between Disease-Modifying Therapies for Multiple Sclerosis and Healthcare Utilisation on a Population Level: A Retrospective Cohort Study. BMJ Open (2019) 9:e033599. doi: 10.1136/bmjopen-2019-033599 PMC6887031 31772108

[11] Pirttisalo AL Sipilä JOT Soilu-Hänninen M Rautava P Kytö V. Adult Hospital Admissions Associated With Multiple Sclerosis in Finland in 2004-2014. Ann Med (2018) 50:354–60. doi: 10.1080/07853890.2018.1461919 29629575

[12] Sanchirico M Caldwell-Tarr A Mudumby P Hashemi L Dufour R. Treatment Patterns, Healthcare Resource Utilization, and Costs Among Medicare Patients With Multiple Sclerosis in Relation to Disease-Modifying Therapy and Corticosteroid Treatment. Neurol Ther (2019) 8:121–33. doi: 10.1007/s40120-018-0123-y PMC6534679 30565050

[13] Simbrich A Thibaut J Khil L Berger K Riedel O Schmedt N. Drug-Use Patterns and Severe Adverse Events With Disease-Modifying Drugs in Patients With Multiple Sclerosis: A Cohort Study Based on German Claims Data. Neuropsychiatr Dis Treat (2019) 15:1439–57. doi: 10.2147/ndt.S200930 PMC6549763 31213818

[14] Statistics Canada. Census Profile, 2016 Census (2017). Available at: https://www12.statcan.gc.ca/census-recensement/2016/dp-pd/prof/index.cfm?Lang=E (Accessed 1 February, 2021).

[15] British Columbia Ministry of Health. Consolidation File (MSP Registration & Premium Billing). V2. Population Data BC [Publisher]. Data Extract. MOH (2017). Available at: http://www.popdata.bc.ca/data.

[16] BC Vital Statistics Agency. Vital Statistics Deaths. V2. Population Data BC [publisher]. Data Extract. BC Vital Statistics Agency (2017). Available at: http://www.popdata.bc.ca/data.

[17] British Columbia Ministry of Health. Medical Services Plan (MSP) Payment Information File. V2. Population Data BC [publisher]. Data Extract. MOH (2017). Available at: http://www.popdata.bc.ca/data.

[18] Canadian Institute for Health Information. Discharge Abstract Database (Hospital Separations). V2 (2017). Population Data BC [publisher]. Data Extract. MOH. Available at: http://www.popdata.bc.ca/data (Accessed (2017)).

[19] BC Ministry of Health. PharmaNet. V2. BC Ministry of Health [Publisher]. Data Extract. Data Stewardship Committee (2017). Available at: http://www.popdata.bc.ca/data.

[20] Kingwell E Zhu F Marrie RA Fisk JD Wolfson C Warren S. High Incidence and Increasing Prevalence of Multiple Sclerosis in British Columbia, Canada: Findings From Over Two Decades (1991–2010). J Neurol (2015) 262:2352–63. doi: 10.1007/s00415-015-7842-0 PMC4608995 26205633

[21] Marrie RA Yu N Blanchard J Leung S Elliott L. The Rising Prevalence and Changing Age Distribution of Multiple Sclerosis in Manitoba. Neurology (2010) 74:465–71. doi: 10.1212/WNL.0b013e3181cf6ec0 20071664

[22] Ng HS Zhu F Kingwell E Zhao Y Yao S Ekuma O. Characteristics of a Population-Based Multiple Sclerosis Cohort Treated With Disease-Modifying Drugs in a Universal Healthcare Setting. Expert Rev Neurother (2020) 21:131–40. doi: 10.1080/14737175.2021.1847085 33146570

[23] Canadian Institute for Health Information and Statistics Canada. Health Indicators 2013 (2013). Available at: https://www.cihi.ca/sites/default/files/document/health-indicators-2013-en.pdf (Accessed 3 March, 2021).

[24] Deyo RA Cherkin DC Ciol MA. Adapting a Clinical Comorbidity Index for Use With ICD-9-CM Administrative Databases. J Clin Epidemiol (1992) 45:613–9. doi: 10.1016/0895-4356(92)90133-8 1607900

[25] Marrie RA Bernstein CN Peschken CA Hitchon CA Chen H Fransoo R. Intensive Care Unit Admission in Multiple Sclerosis: Increased Incidence and Increased Mortality. Neurology (2014) 82:2112–9. doi: 10.1212/WNL.0000000000000495 PMC4118502 24808019

[26] Graham DJ Staffa JA Shatin D Andrade SE Schech SD La Grenade L. Incidence of Hospitalized Rhabdomyolysis in Patients Treated With Lipid-Lowering Drugs. JAMA (2004) 292:2585–90. doi: 10.1001/jama.292.21.2585 15572716

[27] Wallin MT Culpepper WJ Campbell JD Nelson LM Langer-Gould A Marrie RA. The Prevalence of MS in the United States: A Population-Based Estimate Using Health Claims Data. Neurology (2019) 92:e1029–40. doi: 10.1212/WNL.0000000000007035 PMC6442006 30770430

[28] Evans C Zhu F Kingwell E Shirani A van der Kop ML Petkau J. Association Between Beta-Interferon Exposure and Hospital Events in Multiple Sclerosis. Pharmacoepidemiol Drug Saf (2014) 23:1213–22. doi: 10.1002/pds.3667 24953054

[29] Ng HS Zhu F Kingwell E Zhao Y Yao S Ekuma O. Disease-Modifying Drugs for Multiple Sclerosis and Subsequent Health Service Use. Mult Scler (2021) 13524585211063403:1–14. doi: 10.1177/13524585211063403 PMC8958569 34949130

[30] Lin DY Wei LJ Yang I Ying Z. Semiparametric Regression for the Mean and Rate Functions of Recurrent Events. J R Stat Soc Ser B Stat Methodol (2000) 62:711–30. doi: 10.1111/1467-9868.00259

[31] Ballinger GA. Using Generalized Estimating Equations for Longitudinal Data Analysis. Organ Res Methods (2004) 7:127–50. doi: 10.1177/1094428104263672

[32] Cisternas M Bartolome L Gitar B Hulbert E Trenz H Patel V. Health Care Resource Utilization and Disease Modifying Treatment Use in Multiple Sclerosis Patients by Age and Insurance Type. Curr Med Res Opin (2021) 37:597–604. doi: 10.1080/03007995.2021.1885367 33535846

[33] Zhang Y Gonzalez Caldito N Shirani A Salter A Cutter G Culpepper W. 2ndAging and Efficacy of Disease-Modifying Therapies in Multiple Sclerosis: A Meta-Analysis of Clinical Trials. Ther Adv Neurol Disord (2020) 13:1756286420969016. doi: 10.1177/1756286420969016 33552235 PMC7838219

[34] Tramacere I Del Giovane C Salanti G D’Amico R Filippini G. Immunomodulators and Immunosuppressants for Relapsing-Remitting Multiple Sclerosis: A Network Meta-Analysis. Cochrane Database Syst Rev (2015) 9:CD011381. doi: 10.1002/14651858.CD011381.pub2 PMC9235409 26384035

[35] Bonafede MM Johnson BH Watson C. Health Care-Resource Utilization Before and After Natalizumab Initiation in Multiple Sclerosis Patients in the US. Clinicoecon Outcomes Res (2013) 6:11–20. doi: 10.2147/CEOR.S55779 24379685 PMC3872088

[36] O’Connor PW Lublin FD Wolinsky JS Confavreux C Comi G Freedman MS. Teriflunomide Reduces Relapse-Related Neurological Sequelae, Hospitalizations and Steroid Use. J Neurol (2013) 260:2472–80. doi: 10.1007/s00415-013-6979-y PMC3824843 23852658

[37] Giovannoni G Gold R Fox RJ Kappos L Kita M Yang M. Relapses Requiring Intravenous Steroid Use and Multiple-Sclerosis-Related Hospitalizations: Integrated Analysis of the Delayed-Release Dimethyl Fumarate Phase III Studies. Clin Ther (2015) 37:2543–51. doi: 10.1016/j.clinthera.2015.09.011 26526385

[38] Weinstock-Guttman B Galetta SL Giovannoni G Havrdova E Hutchinson M Kappos L. Additional Efficacy Endpoints From Pivotal Natalizumab Trials in Relapsing-Remitting MS. J Neurol (2012) 259:898–905. doi: 10.1007/s00415-011-6275-7 22008873

[39] Tremlett H Zhao Y Rieckmann P Hutchinson M. New Perspectives in the Natural History of Multiple Sclerosis. Neurology (2010) 74:2004–15. doi: 10.1212/WNL.0b013e3181e3973f 20548045

[40] Tremlett H Zhao Y Joseph J Devonshire V. Relapses in Multiple Sclerosis Are Age- and Time-Dependent. J Neurol Neurosurg Psychiatry (2008) 79:1368–74. doi: 10.1136/jnnp.2008.145805 18535026

[41] Ziemssen T Rauer S Stadelmann C Henze T Koehler J Penner IK. Evaluation of Study and Patient Characteristics of Clinical Studies in Primary Progressive Multiple Sclerosis: A Systematic Review. PloS One (2015) 10:e0138243. doi: 10.1371/journal.pone.0138243 26393519 PMC4578855

[42] Baldassari LE Fox RJ. Therapeutic Advances and Challenges in the Treatment of Progressive Multiple Sclerosis. Drugs (2018) 78:1549–66. doi: 10.1007/s40265-018-0984-5 30255442

[43] Faissner S Plemel JR Gold R Yong VW. Progressive Multiple Sclerosis: From Pathophysiology to Therapeutic Strategies. Nat Rev Drug Discov (2019) 18:905–22. doi: 10.1038/s41573-019-0035-2 31399729

[44] Shirani A Zhao Y Petkau J Gustafson P Karim ME Evans C. Multiple Sclerosis in Older Adults: The Clinical Profile and Impact of Interferon Beta Treatment. BioMed Res Int (2015) 451912:1–11. doi: 10.1155/2015/451912 PMC4397470 25922836

[45] Sanofi Genzyme A Division of Sanofi-Aventis Canada Inc. LEMTRADA (Alemtuzumab 12mg/1.2ml) Product Monograph [Online] (2020). Available at: https://www.canada.ca/en/health-canada/services/drugs-health-products/drug-products/drug-product-database.html (Accessed 11 January, 2021).

[46] Rommer PS Zettl UK Kieseier B Hartung HP Menge T Frohman E. Requirement for Safety Monitoring for Approved Multiple Sclerosis Therapies: An Overview. Clin Exp Immunol (2014) 175:397–407. doi: 10.1111/cei.12206 24102425 PMC3927900

[47] Teva Canada Limited. COPAXONE (Glatiramer Acetate Injection 20mg/1ml and 40mg/1ml) Product Monograph [Online] (2018). Available at: https://www.canada.ca/en/health-canada/services/drugs-health-products/drug-products/drug-product-database.html (Accessed August 16, 2021).

[48] Novartis Pharmaceuticals Canada Inc. GILENYA (Fingolimod Capsules 0.25mg and 0.5 Mg) Product Monograph [Online] (2019). Available at: https://www.canada.ca/en/health-canada/services/drugs-health-products/drug-products/drug-product-database.html (Accessed August 16, 2021).

[49] U.S National Library of Medicine ClinicalTrials.gov. Discontinuation of Disease Modifying Therapies (DMTs) in Multiple Sclerosis (MS) (DISCOMS) (2021). Available at: https://clinicaltrials.gov/ct2/show/NCT03073603.

[50] Zhang Y Salter A Jin S Culpepper WJ 2nd Cutter GR Wallin M. Disease-Modifying Therapy Prescription Patterns in People With Multiple Sclerosis by Age. Ther Adv Neurol Disord (2021) 14:1–7. doi: 10.1177/17562864211006499 PMC8020738 33868459

[51] Marrie RA Elliott L Marriott J Cossoy M Blanchard J Tennakoon A. Dramatically Changing Rates and Reasons for Hospitalization in Multiple Sclerosis. Neurology (2014) 83:929–37. doi: 10.1212/WNL.0000000000000753 PMC4153848 25085638

