# Supplementary material for: Disease-Modifying Drug Uptake and Health Service Use in the Ageing MS Population
Source: Front Immunol. 2022 Jan 13;12:794075. doi: 10.3389/fimmu.2021.794075 (PMC8792855; doi:10.3389/fimmu.2021.794075)
Supplement: Supplementary file 1 [file Table_1.docx]

**Supplementary Material**

**Supplementary Table 1** Diagnostic codes for MS and other central nervous system demyelinating diseases

| **Diseases** | **ICD-9 code** | **ICD-10 code** |
| --- | --- | --- |
| Multiple sclerosis | 340 | G35 |
| Optic neuritis | 377.3 | H46 |
| Acute transverse myelitis | 323.82, 341.2 | G37.3 |
| Acute disseminated encephalomyelitis | 323 | G36.9 |
| Demyelinating disease of central nervous system (CNS) unspecified | 341.9 | G37.8 |
| Other acute disseminated demyelination | NA | G36 |
| Neuromyelitis optica | 341.0 | G36.0 |

**Supplementary Table 2** MS-specific disease-modifying drugs approved by Health Canada from 1995 to 2017

| **Individual DMD** | **Brand name & related details** | **Health Canada approval date** | **First or second generation drugs** |
| --- | --- | --- | --- |
| Beta-interferon | Interferon beta-1b [Betaseron®] (0.3 mg/vial) | July 1995 | 1^st^ generation |
|  | Interferon beta-1b [Extavia®] (0.3mg/vial) | November 2009 |  |
|  | Peginterferon beta-1a [Plegridy®] (125mcg/0.5ml); (94mcg/0.5ml); (63 mcg/0.5ml);  (starter pack; 63 µg/0.5ml & 94 µg/0.5ml) | August 2015 |  |
|  | Interferon beta-1a [Avonex®] (30 μG/kit); (30 μG/0.5 ml) | April 1998 |  |
|  | Interferon beta-1a [Rebif®] (initiation pack); (8.8 μG); (11 μG); (22 μG); (44 μG); (66μG); (132μG) | February 1998 |  |
| Glatiramer acetate | Copaxone® (20mg/1 vial); (20mg/1 ml); (40mg/1ml)  Glatect® (20mg/1 ml) | October 1997,  August 2017 | 1^st^ generation |
| Natalizumab | Tysabri® (300mg/15ml) | September 2006 | 2^nd^ generation |
| Fingolimod | Gilenya® (0.5mg capsule) | March 2011 | 2^nd^ generation |
| Dimethyl fumarate | Tecfidera® (120 mg capsule)  Tecfidera® (240 mg capsule) | April 2013 | 2^nd^ generation |
| Teriflunomide | Aubagio® (14 mg tablet) | November 2013 | 2^nd^ generation |
| Alemtuzumab | Lemtrada® (12 mg/1.2ml) | December 2013 | 2^nd^ generation |
| Daclizumab^a^ | Zinbryta® (150mg/ml pre-filled syringe);  (150mg/ml pre-filled pen) | December 2016 | 2^nd^ generation |
| Ocrelizumab | Ocrevus® (300mg/ml) | August 2017 | 2^nd^ generation |

^a^Daclizumab was withdrawn from the market in March 2018 due to safety concerns

**Supplementary Table 3** Disease-modifying drug use during follow-up for persons with multiple sclerosis who reached age 55 years at any time before the study end

| **DMD exposure status, n (%)** | **Number of people who reached age 55 years before study end, n=10,741^a^** |
| --- | --- |
| **Ever exposed to any DMD** | 1,657 (15.4) |
| *First DMD <55 years, and continued DMD ≥55 years* | *706 (6.6)* |
| *First DMD <55 years, and no longer taking DMD once ≥55 years* | *596 (5.5)* |
| *First DMD ≥55 years (and was <55 years old at index date)* | *149 (1.4)* |
| *First DMD ≥55 years (and was ≥55 years old at index date)* | *206 (1.9)* |
| **Never exposed to a DMD** | 9,084 (84.6) |

Key: DMD, disease-modifying drug

^a^ Total cohort size=19,360. Of these, by the study end n=10,741/19,360 (55.5%) had reached their 55th birthday, with n=4,125/10,741 (38.4%) doing so by the index date and n=6,616/10,741 (61.6%) during follow-up.
